# Supplementary material for: Association between low internal health locus of control, psychological distress and insulin resistance. An exploratory study
Source: PLoS One. 2023 May 22;18(5):e0285974. doi: 10.1371/journal.pone.0285974 (PMC10202295; doi:10.1371/journal.pone.0285974)
Supplement: Attachment — Submitted filename: Response to Reviewers.docx [file pone.0285974.s001.docx]

**Respons to Reviewers**

Journal requirements:

When submitting your revision, we need you to address these additional requirements.

1. Please ensure that your manuscript meets PLOS ONE's style requirements, including those for file naming. The PLOS ONE style templates can be found at 
<https://journals.plos.org/plosone/s/file?id=wjVg/PLOSOne_formatting_sample_main_body.pdf> and 
<https://journals.plos.org/plosone/s/file?id=ba62/PLOSOne_formatting_sample_title_authors_affiliations.pdf>

Answer: We have made changes to meet the style requirements.

2. Please provide additional details regarding participant consent. In the ethics statement in the Methods and online submission information, please ensure that you have specified (1) whether consent was informed and (2) what type you obtained (for instance, written or verbal, and if verbal, how it was documented and witnessed). If your study included minors, state whether you obtained consent from parents or guardians. If the need for consent was waived by the ethics committee, please include this information.
If you are reporting a retrospective study of medical records or archived samples, please ensure that you have discussed whether all data were fully anonymized before you accessed them and/or whether the IRB or ethics committee waived the requirement for informed consent. If patients provided informed written consent to have data from their medical records used in research, please include this information.

Answer: In Methods, in the ethics statement, row 112, we have written: All participants signed written informed consent.

3. In your Data Availability statement, you have not specified where the minimal data set underlying the results described in your manuscript can be found. PLOS defines a study's minimal data set as the underlying data used to reach the conclusions drawn in the manuscript and any additional data required to replicate the reported study findings in their entirety. All PLOS journals require that the minimal data set be made fully available. For more information about our data policy, please see <http://journals.plos.org/plosone/s/data-availability>.

"Upon re-submitting your revised manuscript, please upload your study’s minimal underlying data set as either Supporting Information files or to a stable, public repository and include the relevant URLs, DOIs, or accession numbers within your revised cover letter. For a list of acceptable repositories, please see <http://journals.plos.org/plosone/s/data-availability#loc-recommended-repositories>. Any potentially identifying patient information must be fully anonymized.

Important: If there are ethical or legal restrictions to sharing your data publicly, please explain these restrictions in detail. Please see our guidelines for more information on what we consider unacceptable restrictions to publicly sharing data: <http://journals.plos.org/plosone/s/data-availability#loc-unacceptable-data-access-restrictions>. Note that it is not acceptable for the authors to be the sole named individuals responsible for ensuring data access.

We will update your Data Availability statement to reflect the information you provide in your cover letter.

Answer: We have added to the Data Avaliablity statement:

Complete data cannot be made publicly available for ethical and legal reasons according to the Swedish regulations of the “Act concerning the Ethical Review of Research Involving Humans (2006:460)” and the Swedish Ethical Reviews Authority. Public availability would compromise participant confidentiality or privacy. Upon request, a list of codes or meaning units can be made available after removal of details that may risk the confidentiality of the participants. To access such data, please contact the University of Gothenburg, Sahlgrenska Academy, Institute of Medicine, Department of Public Health and Community Medicine/Primary Health Care, Box 453, 40530 Gothenburg, Sweden, (generalpractice@allmed.gu.se) or Bledar Daka (bledar.daka@allmed.gu.se).

Additional Editor comments:

Please, in addition to the corrections in your manuscript, you must respond point by point to the comments of the reviewers.

Another additional comment to that of the reviewers is the need to specify in the title that it is an exproratory study, the following title is suggested: "Association between low internal health locus of control, psychological distress and insulin resistance. An exploratory study"

Answer: Thank you, we agree and have changed the title accordingly.

Reviewer 1

This cross-sectional study examines the relationship between insulin resistance, internal locus of control, and psychological stress in a random sample of individuals from southwestern Sweden. The method and results sections are adequately presented.

Every table should be self-explanatory. Tables 2 and 3 have imprecise titles (to the extent that the footnotes of the two tables indicate which is the outcome variable and which are the adjustment variables, which is unnecessary). A suggestion would be:  Table 2. Association of insulin resistance (HOMA-ir) with psychological distress (PD) and low internal locus of control (IHLC). Similarly, the description of the transformation of estimates to percentages is unnecessary because it has already been covered in the section on data analysis.

Answer: Thank you, we have changed the titles of the table 2 and 3 and the footnotes. The title of table is now “Associations of psychological distress (PD) and low internal health locus of control (IHLC) with insulin resistance (HOMA-ir)”. We decided to write insulin resistance last in the title since that is the outcome variable. We have also removed the description of the transformation of estimates to percentages.

Even though the study is exploratory, it would be helpful to provide more evidence for the hypotheses about the physiological mechanisms behind the links found in the study.

Answer: We have added some text and a reference on the hypothesis about the physiological mechanisms. Row 79-81, and row 334-337.

Reviewer 2

The results presented are interesting, however, the authors report that only 5% of the population they studied met the criteria that decided to associate low internal health locus of control and phychological distress with insulin resistance (HOMA-ir).

Therefore, they decided to add other variables to the mathematical model that they proposed, such as physical activity in a stratified manner, which was not contemplated at the beginning.

The authors decided to add variable by variable to the mathematical model until it stopped giving statistically significant results, so they stopped focusing their article on the initial proposal, to assess the hypothesis that low internal health locus of control (IHLC) and psychological Distress (PD) are associated with insulin resistance.

I believe that the results must be ordered and focus on the initial proposal.

Answer: Thank you for drawing our intention to our unclear description! We have changed the order of the results, in the results (row 159-162, and row 169-254), discussion (row 257-266) and abstract (row 35-48). We added confounders s based on theoretical models to avoid confounding. Knowledge from previous studies on IHLC and physical activity contributed to our decision to continue with the analyses with physical activity.

[Note: HTML markup is below. Please do not edit.]

Reviewers' comments:

Reviewer's Responses to Questions

**Comments to the Author**

1. Is the manuscript technically sound, and do the data support the conclusions?

The manuscript must describe a technically sound piece of scientific research with data that supports the conclusions. Experiments must have been conducted rigorously, with appropriate controls, replication, and sample sizes. The conclusions must be drawn appropriately based on the data presented.

Reviewer #1: Yes

Reviewer #2: Partly

2. Has the statistical analysis been performed appropriately and rigorously?

Reviewer #1: Yes

Reviewer #2: Yes

3. Have the authors made all data underlying the findings in their manuscript fully available?

The [PLOS Data policy](http://www.plosone.org/static/policies.action#sharing) requires authors to make all data underlying the findings described in their manuscript fully available without restriction, with rare exception (please refer to the Data Availability Statement in the manuscript PDF file). The data should be provided as part of the manuscript or its supporting information, or deposited to a public repository. For example, in addition to summary statistics, the data points behind means, medians and variance measures should be available. If there are restrictions on publicly sharing data—e.g. participant privacy or use of data from a third party—those must be specified.

Reviewer #1: No

Reviewer #2: Yes

4. Is the manuscript presented in an intelligible fashion and written in standard English?

PLOS ONE does not copyedit accepted manuscripts, so the language in submitted articles must be clear, correct, and unambiguous. Any typographical or grammatical errors should be corrected at revision, so please note any specific errors here.

Reviewer #1: Yes

Reviewer #2: Yes

5. Review Comments to the Author

Please use the space provided to explain your answers to the questions above. You may also include additional comments for the author, including concerns about dual publication, research ethics, or publication ethics. (Please upload your review as an attachment if it exceeds 20,000 characters)

Reviewer #1: This cross-sectional study examines the relationship between insulin resistance, internal locus of control, and psychological stress in a random sample of individuals from southwestern Sweden. The method and results sections are adequately presented.
Every table should be self-explanatory. Tables 2 and 3 have imprecise titles (to the extent that the footnotes of the two tables indicate which is the outcome variable and which are the adjustment variables, which is unnecessary). Una sugerencia sería: Table 2. Association of insulin resistance (HOMA-ir) with psychological distress (PD) and low internal locus of control (IHLC). Similarly, the description of the transformation of estimates to percentages is unnecessary because it has already been covered in the section on data analysis.
Even though the study is exploratory, it would be helpful to provide more evidence for the hypotheses about the physiological mechanisms behind the links found in the study.

Reviewer #2: The results presented are interesting, however, the authors report that only 5% of the population they studied met the criteria that decided to associate low internal health locus of control and phychological distress with insulin resistance (HOMA-ir).
Therefore, they decided to add other variables to the mathematical model that they proposed, such as physical activity in a stratified manner, which was not contemplated at the beginning.
The authors decided to add variable by variable to the mathematical model until it stopped giving statistically significant results, so they stopped focusing their article on the initial proposal, to assess the hypothesis that low internal health locus of control (IHLC) and psychological Distress (PD) are associated with insulin resistance.
I believe that the results must be ordered and focus on the initial proposal.

6. PLOS authors have the option to publish the peer review history of their article ([what does this mean?](https://journals.plos.org/plosone/s/editorial-and-peer-review-process#loc-peer-review-history)). If published, this will include your full peer review and any attached files.

If you choose “no”, your identity will remain anonymous but your review may still be made public.

**Do you want your identity to be public for this peer review?** For information about this choice, including consent withdrawal, please see our [Privacy Policy](https://www.plos.org/privacy-policy).

Reviewer #1: No

Reviewer #2: No

[NOTE: If reviewer comments were submitted as an attachment file, they will be attached to this email and accessible via the submission site. Please log into your account, locate the manuscript record, and check for the action link "View Attachments". If this link does not appear, there are no attachment files.]

While revising your submission, please upload your figure files to the Preflight Analysis and Conversion Engine (PACE) digital diagnostic tool, <https://pacev2.apexcovantage.com/>. PACE helps ensure that figures meet PLOS requirements. To use PACE, you must first register as a user. Registration is free. Then, login and navigate to the UPLOAD tab, where you will find detailed instructions on how to use the tool. If you encounter any issues or have any questions when using PACE, please email PLOS at [figures@plos.org](mailto:figures@plos.org). Please note that Supporting Information files do not need this step.
